# Comparing risk changes of needlestick injuries between countries adopted and not adopted the needlestick safety and prevention act: A meta-analysis

**DOI:** 10.1017/ice.2021.372

**Published:** 2022-09

**Authors:** Y.S. Ou, H.C. Wu, Y.L. Guo, J.S.C. Shiao

**Affiliations:** 1 School of Nursing, College of Medicine, National Taiwan University (NTU), Taipei, Taiwan; 2 Department of Nursing, Hsin Sheng Junior College of Medical Care and Management, Taoyuan City, Taiwan; 3 Environment and Occupational Medicine, College of Medicine, National Taiwan University (NTU) and NTU Hospital, Taipei, Taiwan; 4 Department of Nursing, National Taiwan University Hospital (NTUH), Taipei, Taiwan; 5 Occupational Health Nursing and Education Association of Taiwan (OHNEAT), Taipei, Taiwan

**Keywords:** needlestick injury, Needlestick Safety and Prevention Act, safety-engineered medical device, risk ratio, meta-analysis

## Abstract

**Objectives:**

To determine whether countries that adopted the Needlestick Safety and Prevention Act (NSPA) achieved a reduced risk of needlestick injuries (NSIs).

**Method:**

In this meta-analysis, 3 international databases (Embase, PubMed, and MEDLINE EBSCO) and 1 Chinese database (Airiti Library) were searched using appropriate keywords to retrieve relevant articles, including multiyear NSI incidences that were published after 2010. The Joanna Briggs Institute Critical Appraisal Checklist for Prevalence Studies was used to evaluate article prevalence. A binary random-effects model was used to estimate risk ratio as summary effect. A log scale was used to evaluate differences in risk ratios of NSIs between countries that adopted versus those that did not adopt the NSPA.

**Results:**

In total, 11 articles were included in the meta-analysis from 9 countries, and NSI incidence rates were surveyed between 1993 and 2016. The risk ratios of NSIs in countries with and without the NSPA were 0.78 (95% CI, 0.67–0.91) and 0.98 (95% CI, 0.85–1.12), respectively, and the ratio of risk ratios was 0.79 (95% CI, 0.65–0.98). Reduction in NSI incidence was more prominent in nurses than in physicians.

**Conclusions:**

Our findings suggest that the mandatory use of safety-engineered medical devices in countries that adopted the NSPA had lower NSI incidence in healthcare workers compared with countries without needlestick safety and prevention regulatory policies. Further studies are needed to develop preventive strategies to protect against NSIs in physicians, which should be incorporated into the standards of care established by national regulatory agencies.

Needlestick injuries (NSIs) are a common occupational hazard among healthcare workers (HCWs); they increase the risk of bloodborne infections such as hepatitis B virus, hepatitis C virus, and human immunodeficiency virus. The use of needles with safety-engineered devices can effectively prevent NSIs.^1–3^ However, only a few countries have implemented the Needlestick Safety and Prevention Act (NSPA), which was first passed by the US Congress in 2000, thus directing the Occupational Safety and Health Administration to revise its bloodborne pathogens standard to require healthcare facilities to provide safety-engineered medical devices (SEMDs) to HCWs.^4^ In this study, to determine the importance and effectiveness of this act, we compared the risk ratio of NSIs between countries that have adopted the NSPA and those that have not. An SEMD is a sharp needle with inherent safety features, such as protection shields and retractable syringes, which are used to prevent NSIs.^5,6^ These features reduce the risk of sharps injuries among HCWs and personnel involved in cleaning sharp boxes.^7^ However, most countries only suggest, rather than mandate, SEMDs due to their high cost,^5^ and few countries have implemented legislation mandating that healthcare facilities provide SEMDs to HCWs.

By 2001, the United States had become the first nation to enact the NSPA, which made mandatory the use of safer medical devices, including sharps with engineered sharps injury protection and needle-less systems, which were meant to reduce or eliminate NSIs among employees.^8^ Between 2010 and 2014, many countries, including the European Union and Taiwan, passed similar legislations that mandate health facilities to provide SEMDs to HCWs to reduce the risk of NSIs. The act emphasizes avoiding the use of unnecessary medical sharps and increasing the use of medical devices with safety-engineered protection mechanisms, such as needle-free devices and SEMDs. Countries that have adopted legislation on the use of SEMDs include Austria, Australia, Belgium, Canada, France, Germany, Italy, the Netherlands, Poland, Portugal, Taiwan, the United Kingdom, and the United States^4^; most of these countries are distributed across North America, Europe, and East Asia.

The legislation has effectively increased the use of SEMDs by HCWs,^9^ thereby reducing their risk of sharps injuries and exposure to infectious blood and body fluids. Conversely, optional application of SEMDs in healthcare facilities without a mandate might not reach a significant level of use.^6,7^ Although legislation effectively reduces sharps injuries, it creates a cost burden on healthcare facilities, to some extent.^10^ We conducted a meta-analysis to determine the risk ratio of NSIs between legislated and unlegislated countries and investigated the effectiveness of the NSPA.

## Methods

### Search strategy

Embase, PubMed, MEDLINE EBSCO, and Airiti Library databases were searched for relevant articles using the search keywords (“needlestick injur*” OR “sharps injur*” OR “percutaneous injur*”) AND (“epidemiology” OR “incidence” OR “prevalence”) in the title or abstract. Because most countries initiated SEMD legislation during 2010–2014, we searched articles published after 2010.

### Criteria for inclusion

Articles with numerators and denominators of a multiyear incidence rate of NSIs among HCWs were included. HCWs were defined as personnel involved in healthcare facilities, including professionals, faculty, students, and support staff. The study population was not limited to a single department or certain experience level in using a sharps device. NSI data were obtained from the NSI report system database to reduce recall bias. Moreover, articles from countries that adopted the NSPA might have been excluded if the study period did not meet the timeframe allowed for the year that the act was passed.

Inclusion criteria were as follows: (1) articles reporting the numerators and denominators of a multiyear incidence rate of NSIs, (2) study population not limited to 1 department, (3) NSI data collected from the report system databases, and (4) articles written in English or Chinese. Exclusion criteria were as follows: (1) legislation articles for which the study timeframe did not include the legislative year and (2) review articles.

After inclusion and exclusion criteria were applied, the full text of each included article was reviewed by 2 researchers. Any disagreement was resolved through mutual discussion or by involving a third researcher. After reaching a consensus, the full text of the selected article was extracted.

### Quality assessment

The quality of the articles was evaluated using the Joanna Briggs Institute (JBI) Critical Appraisal Checklist for Prevalence Studies. The JBI checklist, which was published by Munn et al^11^ in 2015, is an appraisal tool for studies measuring incidence or prevalence. The checklist has 9 questions that assess the sample frame, sample method, sample size, subgroup coverage, reliability, and validity of the measuring tool. A statistical analysis was then performed to evaluate the quality of a study. The questions were scored as “yes,” “no,” “unclear,” or “not applicable.” The checklist facilitated a score or grade for quality; the total number of “yes” responses for each study indicated the study quality.

### Statistical analysis

We used risk ratio as the summary effect of our meta-analysis. The incidence rates of NSIs were calculated using different denominators, including the number of HCWs, full-time equivalents, occupied beds, inpatient days, or sharps purchased.^12^ Because varying incidence rates make it difficult to perform a direct comparison with the global incidence rate, we chose risk ratio as our summary effect.

After we read all of the included articles in detail, we entered the numerator and denominator of each study into the open-source meta-analysis program Review Manager version 5.4.1 (Cochrane, London, UK), which calculated the risk ratio of each article according to the following formula:

Risk ratio = incidence rate after the cutoff year ÷ incidence rate before the cutoff year

Considering the calculation of risk ratios, we set a time point (ie, cutoff year) for each article. For legislation articles, the cutoff year was 1 year after legislation because of the buffering of law. For non-legislation articles, the cutoff year was the middle year of their time frame. For example, for an article with an incidence rate reported from 2010 to 2015, if the data were collected from a legislated country and the year of legislation was 2011, then the cutoff year was 2012. We combined incidence rates of 2010 and 2011 as the incidence rate before legislation, and we combined the data from 2012–2015 as the incidence rate after legislation. If the data were from an unlegislated country, the cutoff year was 2013.

We used a binary random-effects model to estimate summary effects and constructed a forest plot to visually demonstrate results. Heterogeneity was measured by *I* ^2^, and a funnel plot and the Egger test were used to assess publication bias. Finally, a log scale was used to test subgroup differences and to calculate the ratio of risk ratio.^13^

## Results

### Search results

**Fig. 1.**
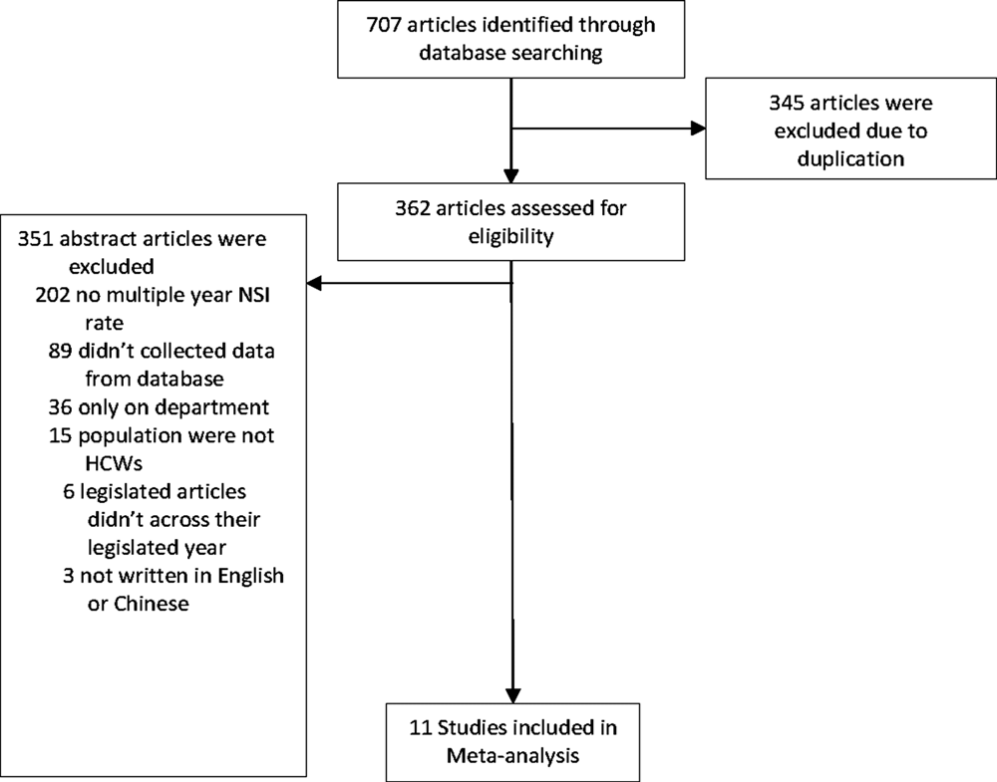
Flowchart of study selection.

Relevant articles were searched from the 4 databases on October 13, 2020. Overall, 292 articles were identified from Embase, 211 from PubMed, 178 from MEDLINE EBSCO, and 26 from Airiti Library. After duplicate studies were eliminated and the abstracts and full text were screened, 11 studies were included in our meta-analysis (Fig. 1).

### Characteristics of included studies

**Fig. 2.**
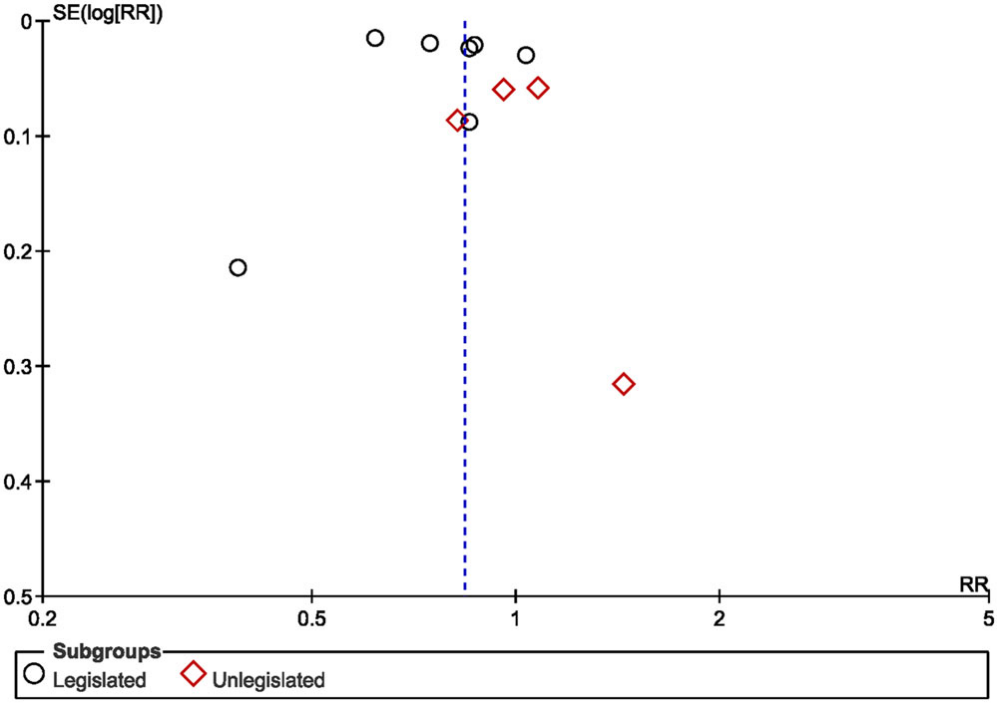
Funnel plot to assess publication bias for relative risk of NSIs.

The 11 included articles were from 9 countries across 1993–2016 (Table 1). Some of the studies collected data from 1 hospital, whereas others collected data from >80 hospitals. Moreover, 7 studies were conducted in legislated countries, for which the cutoff year ranged from 2001 to 2015, whereas 4 studies were conducted in unlegislated countries, for which the cutoff year ranged from 2004 to 2014. Among the studies, 5 used the number of personnel as their denominator of incidence, with an NSI incidence between 0.01 and 0.05. Of these studies, 3 used the number of full-time equivalents (FTE) as the denominator, with an NSI rate between 0.003 and 0.04. The funnel plot (Fig. 2) and the Egger test (*P* = .88) indicated no publication bias.

**Table 1.** Characteristics of Included Studies

Study No.	Study	Participants	Survey Year	Intervention	Cutoff Year	Denominator of NSI	Country
01	Bianco 2019^14^	HCWs of a hospital	1995, 2005, 2015	Legislation	2015	Personnel	Italy
02	Chaiwarith 2013^15^	HCWs of a hospital	2005–2010	…	2008	Personnel	Thailand
03	Chamber 2015^16^	HCWs of Ontario province	2004–2012	Legislation	2009	FTEs	Canada
04	Cheung 2010^17^	Nursing students of a nursing school	2002–2005	…	2004	Personnel	Hong Kong
05	Garus 2018^18^	HCWs of 36 hospitals	2010–2014	Legislation	2014	Personnel	Poland
06	Lee 2017^19^	HCWs of a hospital	2011–2015	…	2014	Person year	Korea
07	Lu 2015^20^	HCWs of 15 healthcare facilities	2006–2010	Legislation	2009	FTEs	Canada
08	Memish 2013^21^	HCWs of 3 hospitals	2007–2011	…	2009	Occupied beds	Saudi Arabia
09	Perry 2012^22^	HCWs of 69 hospitals	1993–1994, 2006–2007	Legislation	2001	Daily census	USA
10	Phillips 2013^23^	HCWs of 85 hospitals	1995–2005	Legislation	2001	FTEs	USA
11	Wu 2019^9^	HCWs of 36 hospitals	2011–2016	Legislation	2013	Personnel	Taiwan

Note. NSI, needle-stick injury; FTE, full-time equivalent.

### Quality assessment

We used the total number of “yes” responses to reflect the quality of each study. Questions 1–5 evaluated the appropriateness of the sample; questions 6 and 7 evaluated the reliability and validity of the measuring tool; question 8 evaluated the statistical analysis; and question 9 evaluated the response rate. The number of “yes” responses of the included studies ranged from 4 to 8, with most studies having a stable report system such as EPINet. Although it is difficult to rule out underreported NSIs, we did not consider them as a “yes” in question 9 because most studies did not examine whether underreporting was a limitation (Table 2).

**Table 2.** Result of Joanna Briggs Institute (JBI) Critical Appraisal Checklist for Prevalence Studies of Included Studies

Item	Study Number Y = Yes and N = No										
	01	02	03	04	05	06	07	08	09	10	11
Was the sample frame appropriate to address the target population?			Y		Y		Y		Y	Y	Y
Were study participants sampled in an appropriate way?			Y	Y	Y	Y	Y	Y	Y	Y	Y
Was the sample size adequate?	Y	Y	Y	Y	Y	Y	Y	Y	Y	Y	Y
Were the study subjects and the setting described in detail?	Y	Y	Y	Y	Y	Y	Y	Y			Y
Was the data analysis conducted with sufficient coverage of the identified sample?	Y	Y	Y	Y	Y	Y	Y	Y	Y	Y	Y
Were valid methods used for the identification of the condition?	Y	Y	Y	Y	Y	Y	Y	Y	Y	Y	Y
Was the condition measured in a standard, reliable way for all participants?	Y		Y	Y	Y	Y	Y	Y	Y	Y	Y
Was there appropriate statistical analysis?	Y		Y	Y	Y	Y	Y	Y	Y	Y	Y
Was the response rate adequate, and if not, was the low response rate managed appropriately?	NA	NA	NA	NA	NA	NA	NA	NA	NA	NA	NA
Total no. of “yes” responses	6	4	8	7	8	7	8	7	7	7	8

Note. NA, not applicable.

### Summary finding of included articles

**Fig. 3.**
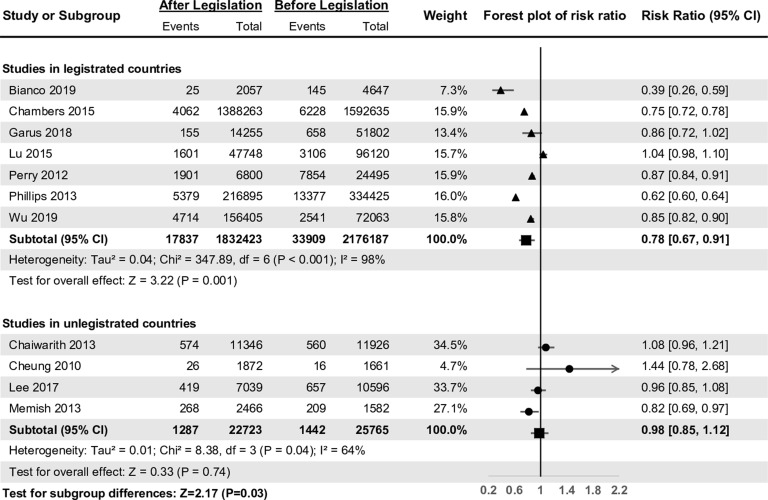
Forest plot of the summary effect analysis among health care workers between legislated and unlegislated countries.

The risk ratio of the 7 articles from legislated countries, assessing the incidence of NSI during 1993–2016, ranged from 0.39 to 1.04. The risk ratio of the 4 articles from unlegislated countries, assessing the incidence of NSI during 2002–2015, ranged from 0.82 to 1.44 (Fig. 3).

### Study effects and subgroup analysis

#### Analysis by HCWs

The risk ratio of the 11 included articles was 0.84, and 95% CI and *I* ^2^ were 0.74–0.96 and 98%, respectively. The risk ratio and *I* ^2^ of 7 articles from legislated countries were 0.78 (95% CI, 0.67–0.91) and 98%, respectively. Except for the study by Lu et al,^20^ 6 other studies showed a lower risk after legislation. Among 4 articles from unlegislated countries, only 1 article showed a significant change in the incidence rate after the cutoff year, and the risk ratio and *I* ^2^ of unlegislated countries were 0.98 (95% CI, 0.85–1.12) and 64%, respectively (Fig. 3). The *Z* test value for subgroup differences was 2.17 (*P* = .03), and the ratio of risk ratio was 0.79 (95% CI, 0.65–0.98).

#### Analysis by job category

**Fig. 4.**
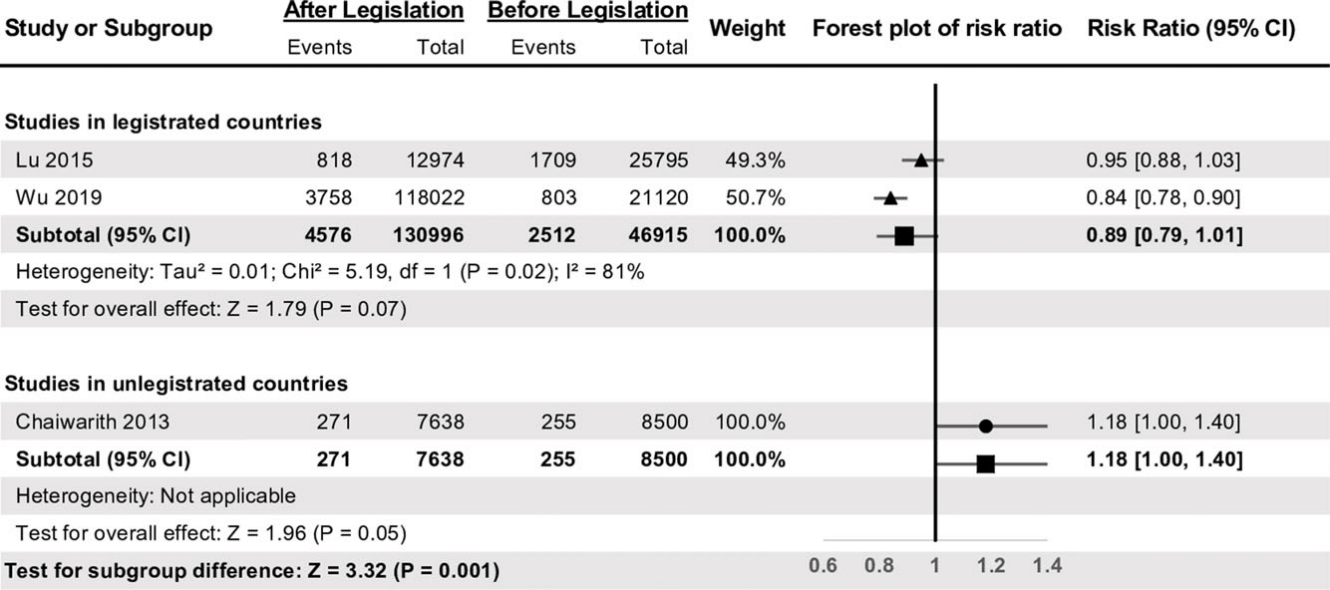
Forest plot of the summary effect analysis among nurses between legislated and unlegislated countries.

In 3 articles, the incidence rates of NSIs were calculated based on job categories of HCWs. We analyzed data from those articles to compare the risk ratio between nurses and physicians. First, we analyzed the data collected from nurses (Fig. 4), and the risk ratio and *I* ^2^ of 3 included articles were 0.96 (95% CI, 0.82–1.13) and ∼90%, respectively. Among legislated countries, the risk ratio was 0.89 (95% CI, 0.79–1.01), and *I* ^2^ was also >80%. The risk ratio of articles from unlegislated countries was 1.18 (95% CI, 1.00–1.40). The *Z* test value for the subgroup difference was 3.32 (*P* = .001), and the ratio of risk ratio was 0.75 (95% CI, 0.62–0.90).

**Fig. 5.**
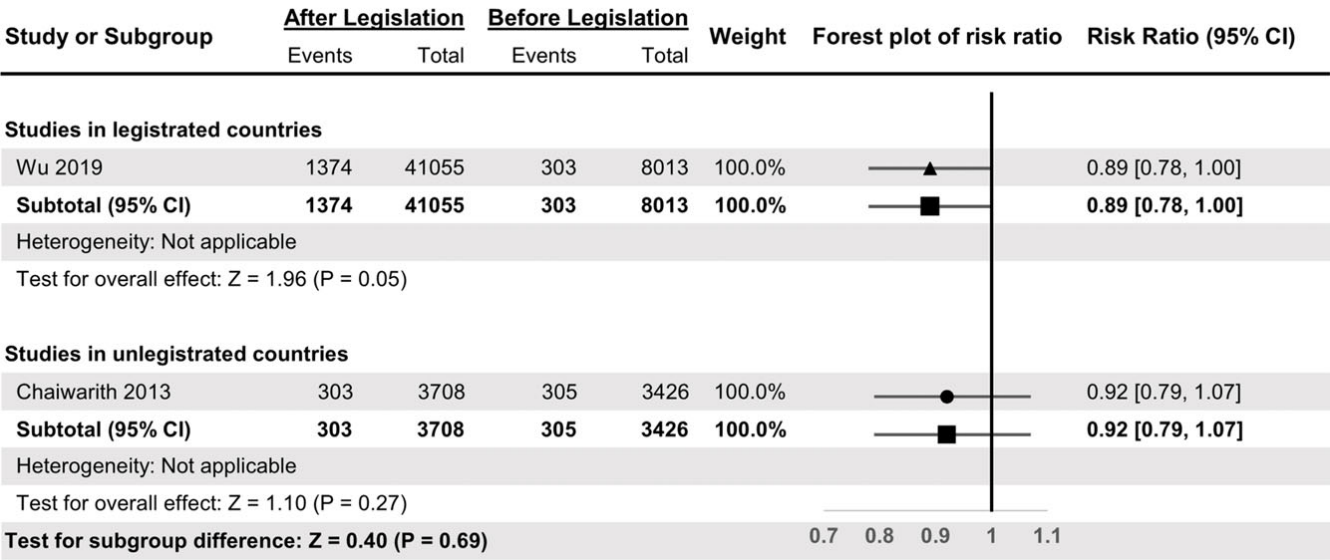
Forest plot of summary effect analysis among physicians between legislated and unlegislated countries.

Only 2 articles calculated NSI incidence among physicians (Fig. 5), and the risk ratio of those articles was 0.90 (95% CI, 0.82–0.99). The risk ratio of the legislated article was 0.89 (95% CI, 0.78–1.00), whereas that of the unlegislated article was 0.92 (95% CI, 0.79–1.07). Because both groups included 1 article only, there were no data regarding heterogeneity. The *Z* test value for the subgroup difference was 0.40 (*P* = .69), and the ratio of risk ratio was 0.75 (95% CI, 0.79–1.17).

## Discussion

The objective of this meta-analysis was to determine whether countries with SEMD legislation achieved reduced NSI incidence. We found that the NSI incidence in legislated countries had decreased significantly and that the reduction significantly differed from that in countries without SEMD legislation. According to 7 articles from legislated countries, the risk of NSIs among HCWs in legislated countries decreased by 22% in 3–6 years after legislation enactment. Articles from unlegislated countries showed no significant change in the NSI incidence among HCWs after 5 years. NSI incidence decreased significantly following SEMD legislation. In Canada, which enacted SEMD legislation in 2009, the NSI incidence rate decreased by 43.3% (from 9.44 to 5.35 per 10,000 FTE) from 2006 to 2011.^16^ In Poland, which enacted SEMD legislation in 2014, the NSI incidence rate did not change significantly from 2010 to 2013 (from 11.55 to 13.82 per 1,000 HCWs), but from 2013 to 2014, the incidence rate decreased by 14.5%. In the United States, which passed the NSPA in 2000, the incidence rate decreased by 38% from 1995 to 2005 (from 4.00 to 2.48 per 100 FTE).^22,23^ In Taiwan, which implemented SEMD legislation in 2012, the NSI incidence rate decreased by 31% (from 3.6 to 2.48 per 100 HCWs). We detected a negative correlation between SEMDs and NSIs; devices with a higher replacement rate of SEMDs had lower NSIs.^9^ In Thailand and Korea, which do not have SEMD legislation, the NSI incidence rate decreased by 8.3% from 2005 to 2010,^15^ and by 16.2% from 2011 to 2015 (from 6.8 to 5.7 per 100 person year),^19^ respectively. Thus, their incidence rates of NSIs had not changed as significantly as it had in Taiwan. The reason for decreases in unlegislated countries may be that some unlegislated countries have begun to use SEMDs; however, widespread use of SEMDs might not be high due to a lack of legal enforcement. Moreover, studies show that significant decreases in NSIs do not usually occur in the 1–2 years after passage of the act.^20^ Another Canadian study found that effectiveness was not achieved until 4 years after SEMD legislation was enacted.^16^

After the enactment of the NSPA, varied effects occurred among different job categories. In our analysis, the risk of NSI incidence in nurses in unlegislated countries increased by 18% after the cutoff year, while an insignificant decrease in NSI incidences occurred in nurses of legislated countries. Nurses were the most frequent users of needles and were also the highest risk group for NSIs before legislation^9,14^; the NSI incidence among Italian and Taiwanese nurses decreased by 68.4% and 32.4% in the 5–10 years after legislation, respectively, and the group with the highest risk of NSIs was replaced by physicians. In unlegislated countries, the NSI incidence among nurses, nurse students, and nurse assistants in Thailand decreased by only ∼15% in 5 years.^15^ Most needles used by nurses were changed from conventional needles to SEMDs after the legislation. The proportion of SEMDs used for intravenous catheters in Taiwan increased from 30% to 93.7% in the 5 years after legislation, and NSIs related to intravenous catheters among nurses decreased from 1.3% to 0.07%.^9^ The NSIs of healthcare assistants also changed. In the study by Bianco et al,^14^ the decline was greatest in healthcare assistants; their risk dropped by 92% after 10 years. Healthcare assistants were not users of sharps, but they might have been exposed to sharps due to incorrect disposal of sharps by other users. Therefore, use of SEMDs can reduce NSIs due to improper disposal of needles, and thus, SEMD legislation can prevent NSIs in healthcare assistants. Study results of Perry et al^22^ showed that the proportion of disposal-related NSIs decreased from 36.8% to 11.6%, and NSIs that occurred during use became the largest proportion of NSIs after legislation, which indicated better protection for assistants and supportive personnel.

We detected no significant decreases in physicians’ NSIs. The differences in the effects of legislation on nurses and physicians could be correlated to differences in the procedures they perform and in the use rate of SEMDs. Our meta-analysis showed that physicians became the most at-risk group after SEMD legislation; therefore, prevention strategies for NSIs were not as effective for physicians as for nurses.^9,14^ Also, 5–10 years after SEMD legislation was enacted, the NSI incidence among nurses decreased by 68.4% and 32.4% in Italy and Taiwan, respectively, but the NSI incidence among physicians decreased only by 48.9% and 12.4%, respectively.^9,14^ The reason for these different effects may be that physicians do not use SEMDs as much as nurses. NSIs among Italian and German physicians were more correlated with conventional sharps, whereas in nurses, NSIs were more correlated with SEMDs.^24–26^ Further study is needed to understand the usage rates of SEMDs based on occupation, which can lead to improved preventive strategies against NSIs in higher-incidence groups.

This study has several limitations. First, the included articles did not mention the coverage rate of SEMDs. Previous studies reported a negative correlation between rates of SEMDs usage and NSI incidence, as higher SEMDs usage lowered the number of NSI incidents.^9,27^ However, few articles examined the relationship between the replacement rate of SEMDs and NSIs incidence among countries that have adopted the NSPA. Second, other policies may have affected changes in the NSI incidence rates. Factors influencing NSI incidents include heavy workload and long work hours^28^; no evidence from this analysis indicated that countries had considered these factors when developing regulations for preventing NSIs. Third, statistical heterogeneity in our results was detected. However, high heterogeneity is common among studies about prevalence and incidence in diverse environments when based on different populations, policy implementation, or time-of-outcome measurements.^11,12,29,30^ We carefully used the random-effects method for data analysis; however, high heterogeneity may lead to issues with interpretability and usefulness than initially anticipated. Last, although the results of the funnel plot and the Egger test showed no publication bias, our inclusion criteria may have resulted in excluding studies from developing or middle-income countries. In addition, we only searched publications from 3 international databases (Embase, PubMed, Medline EBSCO) and 1 Chinese database (Airiti Library), so gray literature that might reflect the real situation in some countries might have been missed. The generalizability of our results might be limited, although the findings might stimulate developing countries to consider adopting SEMDs in healthcare facilities.

In summary, our analysis indicates that NSI incidence in HCWs decreased significantly in countries with SEMD legislation compared with HCWs in countries without SEMD legislation. Further study to determine whether such reductions differ among occupational subgroups can lead to improved regulations.
